# AMP-Activated Protein Kinase Regulates the Cell Surface Proteome and Integrin Membrane Traffic

**DOI:** 10.1371/journal.pone.0128013

**Published:** 2015-05-26

**Authors:** Eden Ross, Rehman Ata, Thanusi Thavarajah, Sergei Medvedev, Peter Bowden, John G. Marshall, Costin N. Antonescu

**Affiliations:** Department of Chemistry and Biology, Ryerson University, 350 Victoria Street, Toronto, Ontario, M5B 2K3, Canada; University of Nebraska Medical Center, UNITED STATES

## Abstract

The cell surface proteome controls numerous cellular functions including cell migration and adhesion, intercellular communication and nutrient uptake. Cell surface proteins are controlled by acute changes in protein abundance at the plasma membrane through regulation of endocytosis and recycling (endomembrane traffic). Many cellular signals regulate endomembrane traffic, including metabolic signaling; however, the extent to which the cell surface proteome is controlled by acute regulation of endomembrane traffic under various conditions remains incompletely understood. AMP-activated protein kinase (AMPK) is a key metabolic sensor that is activated upon reduced cellular energy availability. AMPK activation alters the endomembrane traffic of a few specific proteins, as part of an adaptive response to increase energy intake and reduce energy expenditure. How increased AMPK activity during energy stress may globally regulate the cell surface proteome is not well understood. To study how AMPK may regulate the cell surface proteome, we used cell-impermeable biotinylation to selectively purify cell surface proteins under various conditions. Using ESI-MS/MS, we found that acute (90 min) treatment with the AMPK activator A-769662 elicits broad control of the cell surface abundance of diverse proteins. In particular, A-769662 treatment depleted from the cell surface proteins with functions in cell migration and adhesion. To complement our mass spectrometry results, we used other methods to show that A-769662 treatment results in impaired cell migration. Further, A-769662 treatment reduced the cell surface abundance of β1-integrin, a key cell migration protein, and AMPK gene silencing prevented this effect. While the control of the cell surface abundance of various proteins by A-769662 treatment was broad, it was also selective, as this treatment did not change the cell surface abundance of the transferrin receptor. Hence, the cell surface proteome is subject to acute regulation by treatment with A-769662, at least some of which is mediated by the metabolic sensor AMPK.

## Introduction

Cells interact with their environment through the molecules present on their surface. Cell surface proteins, collectively termed the cell surface proteome, are responsible for many cellular functions including cell adhesion and migration, nutrient uptake, and intercellular signaling. As such, the cell surface proteome must be tightly regulated in order to ensure homeostasis under conditions of cellular and systemic challenges.

Cell surface membrane proteins undergo dynamic traffic between the plasma membrane and intracellular endosomes [1]. As such, the steady-state abundance of proteins at the cell surface is determined by the balance of the rate of endocytosis and exocytosis/recycling of each specific protein [1]. About half of the 590 human kinases control various stages of endomembrane traffic [2], suggesting that the cell surface proteome is the subject of extensive control by various cues; however, much of this regulation remains poorly understood [1].

An emerging regulator of endomembrane traffic is cellular metabolism, and specifically energy stress (e.g. insufficient ATP levels) [1], a condition that requires cells to undertake adaptive processes to ensure homeostasis. A key sensor of cell metabolic state is the heterotrimer AMP-activated protein kinase (AMPK), which is activated by an increase in cellular AMP and ADP level relative to ATP [3]. As such, AMPK becomes activated even upon small changes in AMP/ATP, which can occur in virtually all cell types during a wide range of physiological contexts [4]. Many cells may also experience more dramatic yet less frequent changes in energy availability upon nutrient limitation, as occurs during ischemia or hypoxia [4]. Reactive oxygen species (ROS) activate AMPK by several mechanisms, some of which do not require changes in AMP/ATP [5]. Likewise, nitric oxide (NO) elicits AMPK activation, either by dampening mitochondrial ATP production or by direct control of AMPK activation [6]. AMPK is also activated by many pharmacological agents (e.g. the anti-diabetic agent metformin), dietary compounds (e.g. resveratrol), and hormones (e.g. leptin, thyroid hormone, cannabinoids) [4]. Hence, AMPK activation occurs in many cell types, under a number of physiological, pathophysiological and clinical conditions, thus making AMPK a key sensor of cellular and systemic metabolic stress.

During metabolic stress, the increased binding of AMP to AMPK results in a conformational change that allows sustained phosphorylation of T172 on the alpha subunit by LKB1 or CAMKKβ, thus activating AMPK [4]. The physiological importance of AMPK is evinced by the embryonic lethality of mice with genetic knockouts both α1 and α2 subunits of AMPK [7]. Activated AMPK controls a multitude of cellular processes, in general effecting a reduction in energy expenditure and an increase in nutrient intake, thus allowing maintenance of cellular homeostasis under conditions of reduced energy availability [8]. For example, AMPK controls fatty acid metabolism *via* phosphorylation of acetyl CoA carboxylase [8], controls aerobic glycolysis *via* the activation of HIF-1α [9], controls the formation of tight junctions [10], microtubule dynamics [11], and controls the cell cycle *via* p53 phosphorylation [12].

Activated AMPK also limits energy intensive processes and increases nutrient intake by regulation of cell surface membrane traffic [1]. AMPK activation impairs the internalization of the facilitative glucose transporters GLUT4 in skeletal muscle cells [13] and cardiomyocytes [14], and GLUT1 in a variety of cell types [15]. The resulting increase in cell surface GLUTs increases the rate of glucose uptake, which facilitates the maintenance of ATP homeostasis [16]. AMPK activation increases the internalization of the Na/K-ATPase [1] and also controls the cell-surface membrane traffic of the tight junction protein occulin [17], of the fatty acid transporter CD36 [18] and of the Na+/H+ exchanger NHE5 [19]. The extent of the control of the cell surface proteome by AMPK beyond this small but growing number of proteins is unknown. AMPK might be expected to preferentially exert control over cell surface abundance of proteins that contribute to energy-demanding processes.

Cell migration is an energy demanding process, as it requires actin remodeling and coordinated cell surface and endomembrane traffic. As such, cell migration might be tightly controlled, such that the extent of cell migration may match energy availability. Indeed hypoxia-mediated activation of AMPK reduces cell adhesion in endothelial cells [20] and agents that elicit AMPK activation regulate cell adhesion and migration: berberine [21], AICAR and phenformin [22] or metformin [23] alter cell migration. As many of these agents and treatments have cellular effects additional to the activation of AMPK [24], the possible regulation of cell adhesion and migration by AMPK activation requires further study.

Cell adhesion and migration are controlled by the regulated membrane traffic of integrins, a family of transmembrane proteins that physically bridge the actin cytoskeleton to the extracellular matrix. Integrins are heterodimers comprised of one α- and one β-subunit [25]. β1-integrin is the principal binding partner of many α-integrins and as such is a key cell adhesion and migration molecule [25]. The leading edge of the lamellipodium of migrating cells is a zone of dynamic actin remodeling, which generates pushing forces on the membrane, in part as a result of the interaction of integrins with actin filaments [26]. Cell migration requires dynamic integrin membrane traffic [27]. Integrins undergo internalization *via* both clathrin-dependent and-independent mechanisms [28], and are recycled back to the plasma membrane via Rab4, Rab11 and/or Rab21 endosomes [27,29–31]. Hence, the control of integrin membrane traffic regulates cell migration [27].

Whether AMPK may broadly and acutely control the cell surface proteome in order to limit energy expenditure is poorly understood. Recently, methods have been developed to systematically study the cell surface proteome. Several studies have utilized cell-impermeable lysine- or glycan-reactive biotinylation reagents to label surface-exposed proteins, purification of biotinylated proteins followed by protein identification by mass spectrometry. These methods have been useful in performing comparative cell surface proteomics of stem cells of various lineages [32], as well as of human mesenchymal stromal cells [33], showing unique cell surface proteomes of these different cells. Moreover, examination of human mesenchymal stem cells before and after long-term stimulation with basic fibroblast growth factor (bFGF) allowed identification of many proteins that exhibit altered abundance by this treatment [34]. Several studies have specifically scrutinized the cell surface proteome of cancer cells to identify unique features that may be therapeutic targets [35–37].

These studies have revealed unique and common features of the cell surface proteome of various cell types and/or of cells after long-term stimulation with hormones and as such likely reflect changes in protein expression. However, the cell surface abundance of proteins can be regulated acutely, independently of changes in protein expression. This occurs as a result of regulation of membrane traffic for integral membrane proteins and/or of membrane binding for membrane-associated proteins [1]. Indeed systematic analysis of Kc167 cells stimulated with lipopolysaccharide (LPS), rapamycin, vanadate or insulin (for 1–2 h) [38] and MIN-6 cells stimulated with glucagon-like peptide (GLP-1) and 20 mM glucose for 1 hour [39] revealed robust but selective changes in the cell surface proteome by these treatments. These studies demonstrate the power of systematic analysis of the cell surface proteome, and begin to characterize how cell surface proteins are controlled by both acute and chronic stimuli.

In this study, we have used a cell-surface biotinylation strategy to purify cell surface proteins, and analyzed the cell surface abundance of specific proteins in cells acutely stimulated with AMPK activators to that of unstimulated cells using electrospray ionization tandem mass spectrometry (ESI-MS/MS). We find robust control of the cell surface abundance of diverse proteins by AMPK activation, in particular proteins annotated for a function in cell adhesion and migration. By combining this mass spectrometry approach with other methods, we conclude that AMPK controls cell migration and integrin membrane traffic.

## Results

To understand how AMPK may regulate the cell surface proteome, we used an approach coupling selective purification of cell surface proteins with mass spectrometry to compare the cell surface abundance of proteins in cells with activated AMPK to that of control (unstimulated) cells. Selective labeling and purification of cell surface proteins was achieved by use of an amine-reactive, cell-impermeable, cleavable biotinylation reagent, sulfo-NHS-SS-Biotin. This allowed subsequent purification of cell surface proteins using streptavidin-conjugated beads, and elution of purified proteins with a reducing agent. Treatment of cells with sulfo-NHS-SS-Biotin followed by this biotin purification strategy resulted in recovery of a large number of proteins with very few visible protein bands observed in samples from cells not treated with sulfo-NHS-SS-Biotin (background binding to beads), as assessed by silver staining (S1A Fig).

To validate the use of this method to selectively purify cell surface proteins, we probed these purified cell surface samples with antibodies to various specific proteins (S1B Fig). We observed that the epidermal growth factor receptor (EGFR) is found virtually exclusively in the cell surface fraction of cells treated with sulfo-NHS-SS-Biotin. In contrast, the intracellular protein clathrin heavy chain (CHC) was not purified in the cell surface fraction in cells treated with sulfo-NHS-SS-Biotin, despite harboring 94 lysine residues. Two additional cytosolic soluble proteins, actin and Erk, were found predominantly within the intracellular fraction, although they were also detected at modest levels in the cell surface fraction. Thus, Erk and actin, but not CHC, have sufficiently stable association with integral membrane proteins to be detected in the cell surface fraction. Hence, purification of proteins biotinylated by this strategy yields both integral cell surface membrane proteins as well as cytosolic proteins associated with membranes.

### Mass spectrometry analysis of protein content of the cell surface

**Fig 1:**
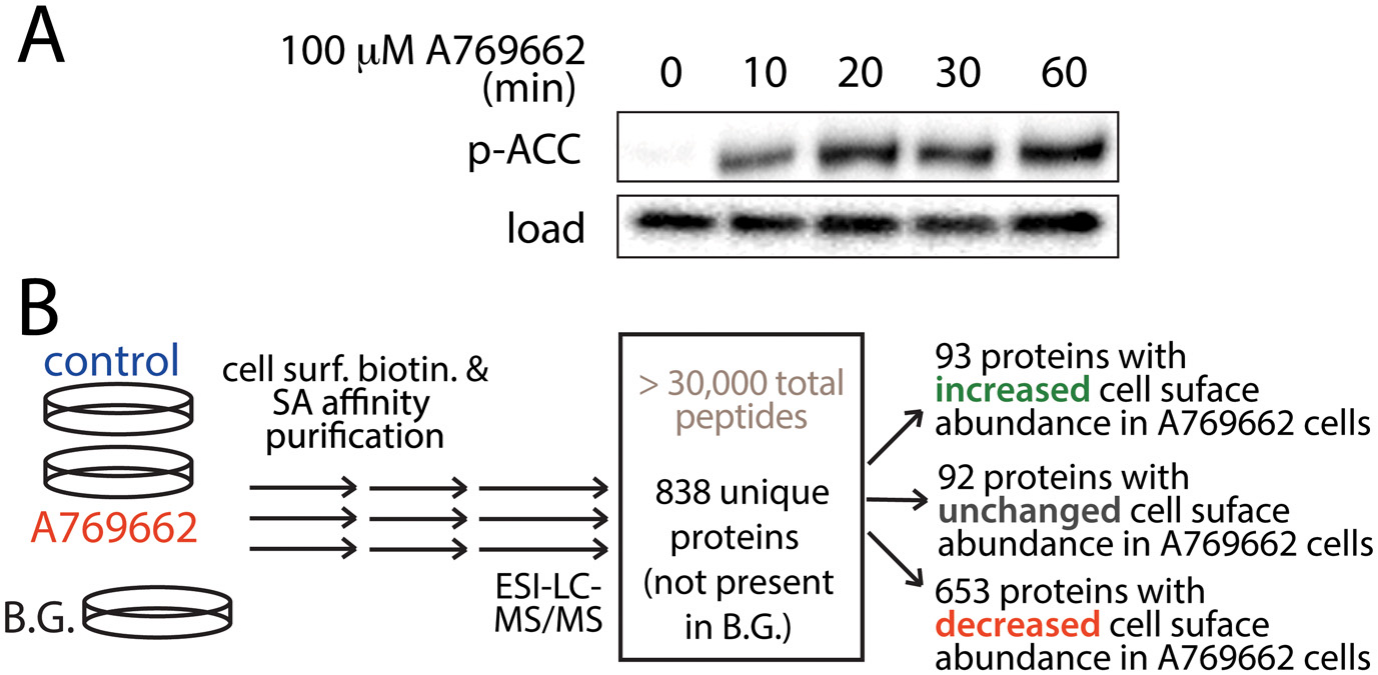
AMPK activation and mass spectrometry analysis of cell surface proteins. (***A***) RPE cells were stimulated with 100 μM A-769662 in media containing 0.1% FBS for indicated times. Shown are representative immunoblots using antibodies as indicated. (***B***) Shown is a diagram depicting cell stimulation, surface biotinylation, purification of biotinylated proteins, mass spectrometry and peptide identification. We thus identified a total of 838 proteins within all cell surface fractions, of which 653 exhibited reduced detection in the cell surface fraction of cells treated with A-769662, 93 proteins exhibited increased cell surface abundance in cells treated with A-769662, and a further 92 were classified as exhibiting largely unaltered detection in the cell surface fraction upon AMPK activation. A complete list of identified proteins can be found in S1 Table.

To investigate the regulation of the cell surface proteome by AMPK, we used ARPE-19 cells (RPE henceforth) as a model. In contrast to many other cell line models, RPE cells are not immortalized [40], a modification which alters cell metabolism and AMPK activation [41,42], which thus may in turn alter or mask cell responses to energy stress signaling. While RPE cells can be differentiated into monolayers that over ~ 1 month develop markers of retinal epithelia, all of our studies focused on undifferentiated RPE cells that exhibit proliferation and migration; in this context RPE cells are an emerging model to study endomembrane traffic [43]. To activate AMPK, we treated cells with A-769662, a well characterized AMPK activator [44] which exhibits fewer off-target effects than other agents that activate AMPK such as 5-aminoimidazole-4-carboxamide ribonucleotide (AICAR) or phenformin [24]. Stimulation of RPE cells with 100 μM A-769662 resulted in a robust increase in AMPK activity, assessed by phosphorylation of the AMPK substrate acetyl CoA carboxylase (ACC) (Fig 1A). The phosphorylation of ACC was visible as early as 10 min upon addition of A-769662.

To investigate the regulation of the cell surface proteome by AMPK activation, we coupled treatment with 100 μM A-769662 for 90 min with cell-surface protein biotinylation and purification. This time-point was chosen to allow any possible AMPK-dependent changes in membrane traffic phenomena to establish new steady-state cell-surface protein levels, while minimizing the contribution of altered whole-cell protein expression or degradation, which may occur over longer periods of time. We prepared three distinct samples that were subjected to purification of the cell surface fraction **(** Fig 1B): 1) control (unstimulated) cells treated with sulfo-NHS-SS-Biotin (“control”), 2) cells first treated with 100 μM A-769662 for 90 min, then with sulfo-NHS-SS-Biotin (“A-769662”), and 3) cells not treated with sulfo-NHS-SS-Biotin (“Background”, or “B.G.”). Following elution of proteins by reduction, proteins were subjected to tryptic digestion and LC-ESI-MS/MS. The experiment was performed 5 times (n = 5), allowing identification of > 30000 peptides, and protein identifications within cell-surface fractions required a minimum of 4 peptide matches. Samples of MS/MS fragment spectra are shown in S2 Fig. Proteins detected in the “Background” condition represent molecules that were present in the cell surface fraction due to non-specific interactions. As such, all proteins that had at least 1 peptide identified in the background sample were discounted from the list of proteins detected in the control and A-769662 conditions; after this process a total of 838 proteins were thus identified as *bona fide* proteins within the cell surface fraction (Fig 1B).

The relative number of peptides of a protein detected by mass spectrometry within different samples scales with protein abundance within each sample; indeed the measurement of the number of peptides detected per protein reflects the relative abundance of that protein in a particular sample [45–47]. This type of analysis using relative peptide identification counting was used previously to quantify the difference in the abundance of specific proteins between biochemically-isolated secretory membrane compartments fractions [48] and between clathrin-coated vesicle proteins isolated from brain *versus* liver [49].

Based on this principle, we used an arbitrary yet stringent method to classify the proteins as depleted or enriched upon A-769662 treatment within the cell surface fraction. We used the same threshold for identification of proteins specific to the cell surface fraction (minimum of 4 peptides identified in cell surface fractions, no peptide identifications in the Background sample) to classify protein enrichment in basal *versus* A-769662-treated cell surface fractions. We classified proteins as being “depleted from the cell surface in A-769662 cells” if we detected a minimum of 4 peptides in the control condition and no (0) peptides corresponding to this protein in the A-769662-treated condition (653 proteins). We classified proteins as being “enriched at the cell surface in A-769662 cells” if we detected a minimum of 4 peptides in the A-769662-treated condition and no (0) peptides corresponding to this protein in the control condition (93 proteins). Finally, proteins with at least 4 total peptide identifications and at least one peptide detected in each of the control and A-769662-treated conditions were classified as “unchanged in cell surface abundance in A-769662 cells” (92 proteins). The complete list of proteins identified in each of these classifications can be found in S1 Table.

Previous studies that characterized the cell surface proteome identified ~100–200 *bona fide* integral membrane proteins [33,38], hence our identification of 838 proteins total cell surface proteins from RPE cells appears to exceed the number of integral membrane proteins identified in these previous studies. We thus examined how many of the cell surface proteins that we identified were integral membrane proteins. Of the 838 total proteins that we identified within the cell surface fraction, 650 had Swiss-Prot (SP) and Protein Information Resource (PIR) Keywords annotations (SP-PIR-KEYWORD). Of these 650 proteins, 100 were annotated as being transmembrane proteins (15.4%). Of the proteins depleted from the cell surface by treatment with 100 μM A-769662, 79 of 592 with SP-PIR-KEYWORD annotations were transmembrane proteins (13.3%). This proportion of integral membrane proteins within the cell surface fraction is similar to other studies that isolated the cell surface proteome of mesenchymal stromal cells, which had identified 169 out of 888 total proteins in the cell surface fraction as integral membrane proteins [33].

Our analysis uncovered that several proteins not readily predicted to associate with the cell surface were indeed present in the cell surface fraction. For example, we identified that ZNF142, a predicted transcription factor based on harboring several C2 zinc finger domains (typically found in DNA-binding transcription factors) was found at the cell surface in basal (74 peptides identified) but not A-769662-treated cells (0 peptides identified). It should be noted that while ZNF142 is predicted to be a transcription factor, we are not aware of any studies to date that have examined the cellular localization or function of this protein. Using immunofluorescence microscopy, we confirmed that ZNF142 localizes mostly outside of the nucleus (S3A Fig). Importantly, in the basal condition, ZNF142 localizes to the cell periphery, which is consistent with association with structures at or near the plasma membrane. Treatment with A-769662 results in loss of the peripheral localization of ZNF142 (S3B Fig), likely reflecting a decreased association of ZNF142 with or near the plasma membrane upon A-769662 treatment.

Hence, while intrinsic membrane proteins can be difficult to solubilize for analysis with mass spectrometry, our method allowed us to monitor both transmembrane and membrane-associated proteins. As regulation of cell surface function occurs as a result of control of cell surface abundance of integral membrane proteins as well as by control of membrane association of proteins lacking a *bona fide* transmembrane domain, this methodology provides a powerful tool to systematically understand the regulation of the cell surface proteome.

### Gene ontology clustering reveals cellular processes regulated by AMPK activation

To identify with high confidence the cellular processes that are regulated by control of protein abundance at the cell surface upon A-769662 treatment, we employed a functional annotation classification approach, using DAVID [50]. This tool measures the enrichment of particular Gene Ontology (GO) annotation terms within a subset of proteins (e.g. “depleted from the cell surface in A-769662 cells”, 653 proteins) given a total list of proteins (e.g. the 838 total proteins detected within all cell surface fractions). This allows for interpretation of MS results at the biological module level [50], and is advantageous to monitor how AMPK regulates the surface proteome by reducing the likelihood that spurious identifications or categorizations influence the findings.

Using the GO functional analysis, we found several functional clusters significantly down-regulated from the cell surface by A-769662 treatment (Table 1). This analysis suggests that proteins involved in regulation of apoptosis, of cell signaling, of cell adhesion and migration, and of the cytoskeleton are significantly (p < 0.05) depleted from the cell surface fraction upon A-769662 treatment.

**Table 1:** Gene Ontology terms depleted from the cell surface upon A-769662 treatment.

GO Term	GO identification	Protein number	Fold Enrichment	p-value
***Apoptosis***				
induction of apoptosis by extracellular signals	GO:0008624	8	5.44	7.68E-05
positive regulation of apoptosis	GO:0043065	21	2.86	6.36E-06
***Cell signaling***				
cell-cell signaling	GO:0007267	15	2.42	0.00162
protein kinase cascade	GO:0007243	12	2.23	0.0119
regulation of cell proliferation	GO:0042127	20	1.73	0.0155
***Cell Adhesion and migration***				
cell adhesion	GO:0007155	32	1.43	0.0286
biological adhesion	GO:0022610	32	1.43	0.0286
***Cytoskeleton***				
cell projection organization	GO:0030030	21	1.95	0.00287
cytoskeleton organization	GO:0007010	20	1.76	0.0132

Shown are the GO terms found to be significantly depleted from the cell surface fraction upon A-769662 treatment, determined by DAVID [50]. For each GO term, shown are the number of proteins detected at the cell surface in control cells, as well as the fold enrichment of proteins with that particular GO classification detected the cell surface fraction of control (resting) cells *vs* all cell surface proteins detected in this study (and corresponding p-value for this enrichment).

As AMPK is known to down-regulate energy intensive processes during energy stress [8], and that cell migration is such a cell intensive process, we focused on the regulation of cell surface abundance of adhesion and migration proteins upon A-769662 treatment. We found that 32 proteins with cell adhesion and migration GO annotations were identified within the “depleted from the cell surface in A-769662 cells” group as a result of at least 4 detected peptides in control cells, with 0 peptide detections in cells treated with A-769662 (n = 5, Table 2). The concerted behaviour of many proteins with similar function strongly indicates that the observation of depletion of cell adhesion and migration from the cell surface upon A-769662 treatment is not simply due to sampling error.

**Table 2:** Proteins with cell adhesion and migration GO classification depleted from the cell surface upon A-769662 treatment.

Gene symbol	Protein description	Peptides detected in basal cond.	Peptides detected in A-769662 cond.	Peptides detected in background
FAT1	FAT tumor suppressor 1	**8**	0	0
FAT2	FAT tumor suppressor 2	**6**	0	0
FAT4	FAT tumor suppressor 4	**7**	0	0
LYPD3	LY6/PLAUR domain containing 3	**7**	0	0
NELL1	PKC binding protein NELL1	**5**	0	0
NELL2	PKC binding protein NELL2	**4**	0	0
SSPO	SCO-spondin	**7**	0	0
CXCL12	chemokine (C-X-C motif) ligand 12	**4**	0	0
COL6A3	collagen, type VI, alpha 3	**6**	0	0
COL112	collagen, type XI, alpha 2	**4**	0	0
COL14A1	collagen, type XIV, alpha 1	**6**	0	0
DLG1	discs, large homolog 1	**5**	0	0
FER	fer (fps/fes related) tyrosine kinase	**4**	0	0
FERMT3	fermitin family homolog 3	**11**	0	0
FNDC3A	fibronectin type III domain cont. 3A	**6**	0	0
ITGA11	integrin, alpha 11	**14**	0	0
ITGA4	integrin, alpha 4	**4**	0	0
ICAM5	intercellular adhesion molecule 5	**5**	0	0
LOXL2	lysyl oxidase-like 2	**5**	0	0
MUC16	mucin 16, cell surface associated	**15**	0	0
MUC5AC	mucin 5AC,	**4**	0	0
NRP1	neuropilin 1	**4**	0	0
PKHD1	polycystic kidney & hepatic disease 1	**5**	0	0
PCHD19	protocadherin 19	**5**	0	0
PCHD19	protocadherin 7	**4**	0	0
PCDHB10	protocadherin β 9	**4**	0	0
PCDHGB3	protocadherin gamma subfamily B, 3	**6**	0	0
SEMA5B	semaphorin 5A	**5**	0	0
SDK1	sidekick homolog 1	**20**	0	0
SORBS1	sorbin and SH3 domain containing 1	**15**	0	0
SNED1	sushi, nidogen & EGF-like domains 1	**4**	0	0
TRO	trophinin	**6**	0	0

Shown are the 32 proteins with Cell Adhesion and Migration GO classification detected in the cell surface fraction of control but not A-769662 treated cells. Shown for each are the detected peptide counts in each of the treatment conditions.

We quantified the results by the peptide to protein counts detected that show the proteins were detected multiple times on the cell surface in resting (control) cells but not in A-769662 treated cells or background controls. Furthermore we statistically analyzed the intensity distribution of the detected peptides to ensure that they showed the expected homogenous population distribution that was separated by at least 2.5 quantiles from the activated cells and therefore could not merely reflect small differences between the detected and undetected proteins. As described previously, when parent ion and MS/MS fragment intensities exhibit a log-normal distribution, differences in peptide/protein measurements between treatment conditions reflect significant differences in protein content [51–53]. We thus analyzed the parent ion and MS/MS fragment intensities of a subset of the cell migration and adhesion proteins that we detected in the control samples but not in the A-769662-treated samples (classified as “depleted from the cell surface in A-769662 cells”): integrin α-11, integrin α-4, ICAM5, collagen 6A3, collagen 14A1, FAT1 and FAT2) **(** S4 Fig). This analysis revealed that log intensity values of the parent ions and MS/MS fragments indeed exhibited a near normal distribution. The proteins were well detected with a high signal-to-noise ratio in the control cell-surface fraction, but were entirely undetectable in the cell surface fraction from A-769662-treated cells, even at very low signal-to-noise levels past two and a half quantiles below the mean of the intensity distribution. This reflects a significant difference in intensity, indicating a significant difference in the abundance of the proteins between control and A-769662-treated cells.

We further excluded the possibility that differences in the detection of proteins between treatments might have occurred as a result of systematic differences in total protein content between treatment samples or sampling errors. We analyzed the distribution of parent ion and peptide fragment detection intensities across treatment conditions of proteins classified as “depleted from the cell surface in A-769662 cells”, “enriched at the cell surface in A-769662 cells”, “unchanged at the cell surface in A-769662 cells” (as per Fig 1 and S1 Table) or that were also found in the background sample. As expected, the proteins classified as “depleted from the cell surface in A-769662 cells” exhibited mean peak intensity values between 0.7–1.0 intensity counts (log scale) within the control samples (with S.E. < 0.05 in each), with no detection of these peptides in the background or AMPK-activated condition samples (S2 Table). The depletion of these proteins from the cell surface by A-769662 was specific, as the proteins classified as “enriched at the cell surface in A-769662 cells” also had peak intensity values between 0.7–1.0 intensity counts (log scale) within the A-769662 treated samples (with S.E. < 0.05 in each), with no detection of these peptides in the background or control samples (S2 Table). Further, a number of fragments corresponding to other proteins were detected with similar intensities in each of the conditions examined (S2 Table). Moreover, the total mean log intensity values detected for parent ions and for MS/MS fragments were not different between each of the treatment conditions (comparing total parent ions and fragments, regardless of protein identification, S3 Table).

Collectively, this analysis of the intensity distribution of peptides and MS/MS fragments strongly indicates that differences in the detection of specific proteins in the cell surface fraction between different treatments (e.g. control *vs*. A-769662-treated samples) was due to a significant difference in the abundance of these proteins in each sample. As such, the selective and concerted depletion of cell migration and adhesion proteins from the cell surface fraction observed upon A-769662 treatment occurred as a biological response to this treatment.

**Fig 2:**
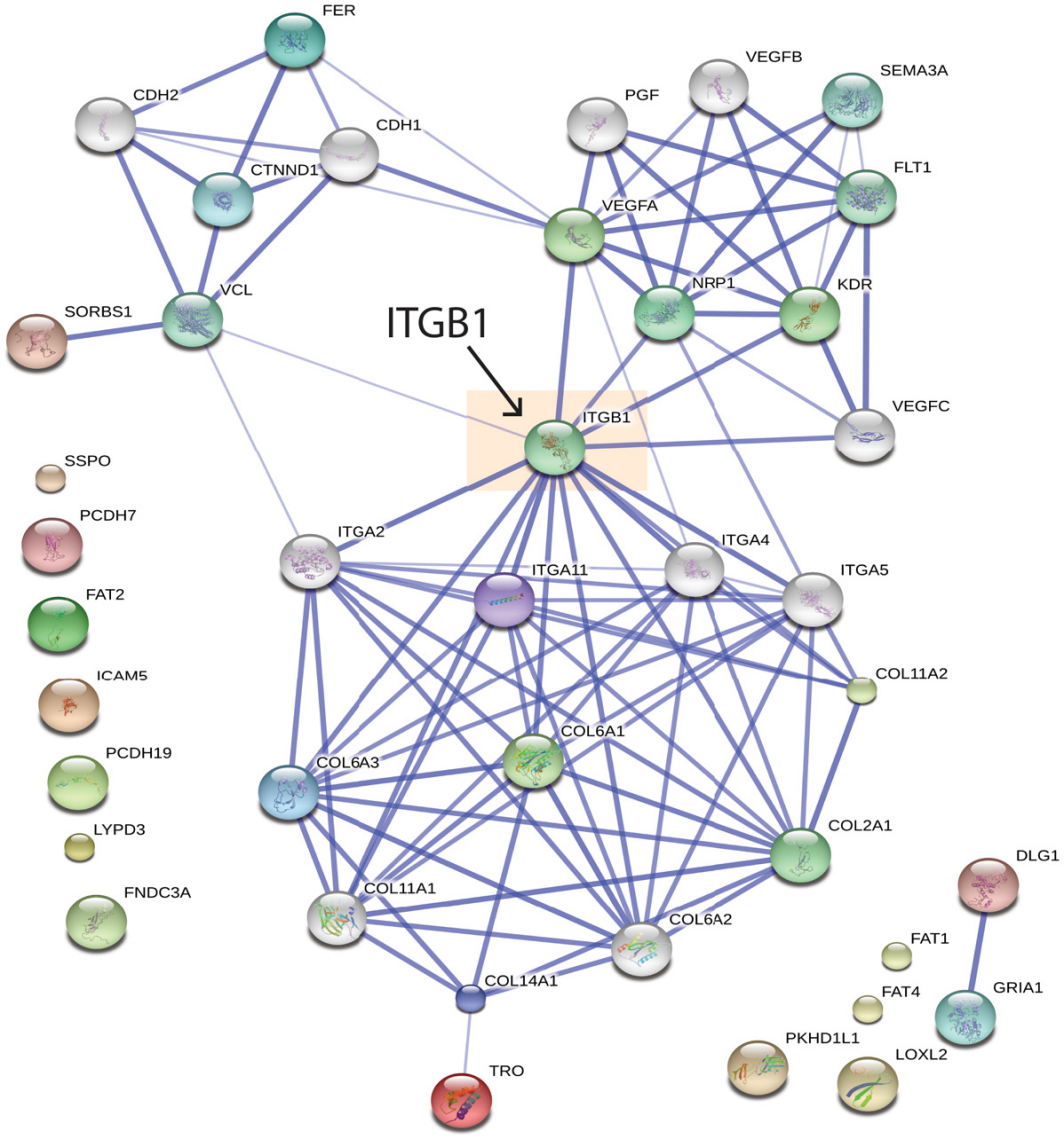
STRING analysis reveals functional interaction of cell migration and adhesion proteins depleted from the cell surface by A-769662 treatment. The list of 32 proteins with cell adhesion and migration GO annotation (Table 2) was subjected to STRING analysis to visualize known and predicted interactions [54]. Shown is a graphical representation of the output of this analysis. Highlighted is the predicted interaction of proteins identified as depleted from the cell surface fraction upon A-769662 treatment with β1-integrin (ITGB1).

To identify how the 32 cell adhesion and migration proteins that were found to be decreased in abundance at the cell surface upon A-769662 treatment may be functionally linked, we performed STRING analysis, which illustrates known and predicted functional interactions within a protein list [54]. STRING analysis of these 32 proteins identified several functionally linked nodes of proteins **(** Fig 2). Importantly, this analysis predicts with high confidence a functional interaction of several of these cell adhesion/migration proteins depleted from the cell surface by A-769662 treatment with β1-integrin (ITGB1). These results predict that A-769662 treatment may alter cell adhesion and cell migration in a manner that involves decreased cell surface abundance of β1-integrin.

### Regulation of cell migration by A-769662 treatment

**Fig 3:**
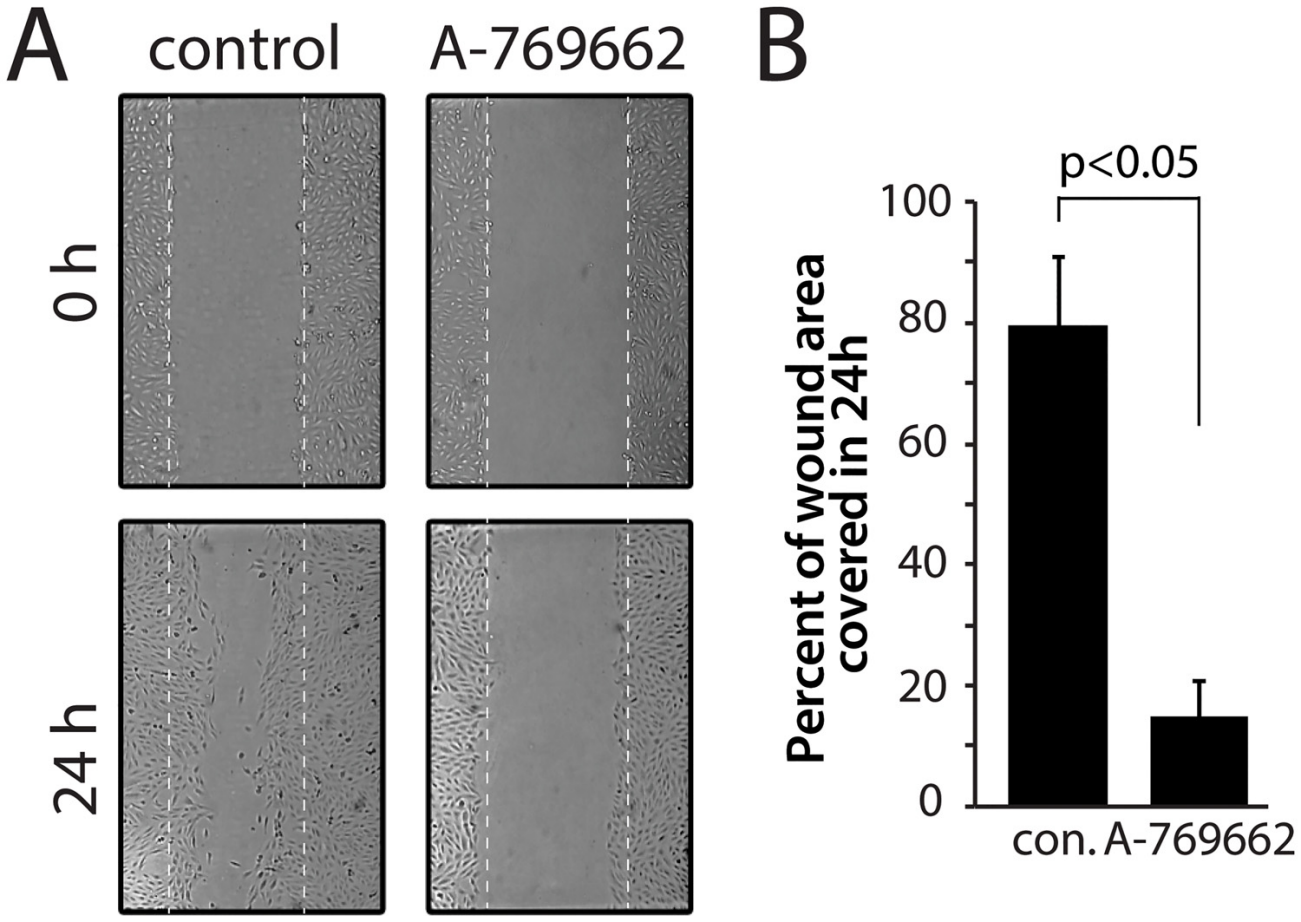
Treatment with A-769662 reduces cell migration. RPE cells were subjected to an epithelial wounding cell migration assay, either under conditions of continuous stimulation with 100 μM A-769662 or unstimulated (control). (***A***) Shown are representative micrographs of cells immediately after wounding (0 h) or 24 h after wounding, as indicated, with the region of the wound indicated by dashed white lines. (***B***) The coverage of the wounded area by cells 24 hours after wounding was quantified; shown are the means ± SE of the percent of wounded area covered by migrating cells in 24h in control and A-769662 treated cells (n = 3, p < 0.05).

Using mass spectrometry approaches, we have identified that A-769662 treatment elicits a reduction of the abundance of cell adhesion and migration molecules at the cell surface. To determine if A-769662 treatment regulates cell migration, we used an epithelial wound-healing assay to monitor cell migration, as had previously been used for RPE cells [55]. These experiments were performed in minimal serum conditions to minimize cell proliferation during the assay. We quantified the fraction of the area of the initial wound that became covered with cells after 24 hours. Wounding of an RPE cell confluent monolayer resulted in cell migration to effect 79.4 ± 11.6% coverage of the wounded area within 24h under control conditions (Fig 3A and 3B). In stark contrast, cells treated with 100 μM A-769662 exhibited greatly reduced cell migration in this assay, such that only 15.5 ± 5.7% of the wounded area was covered in 24h (at least 20 wounds measured per experiment, n = 3, p < 0.05) (Fig 3A and 3B). Cells treated under the conditions used to measure cell migration (0.1% FBS + 100 μM A-769662) did not exhibit positive staining with propidium iodide (S5A Fig), indicating that changes in cell migration upon treatment with A-769662 were unlikely to be caused by a reduction in cell viability. These results indicate that cells treated with A-769662 exhibited reduced cell migration. These finding are consistent with the observations made by mass spectrometry that cell adhesion/migration proteins (including integrins) are reduced in abundance at the cell surface upon A-769662 treatment (Table 2).

### AMPK activation elicits a specific reduction in cell surface β1-integrin

**Fig 4:**
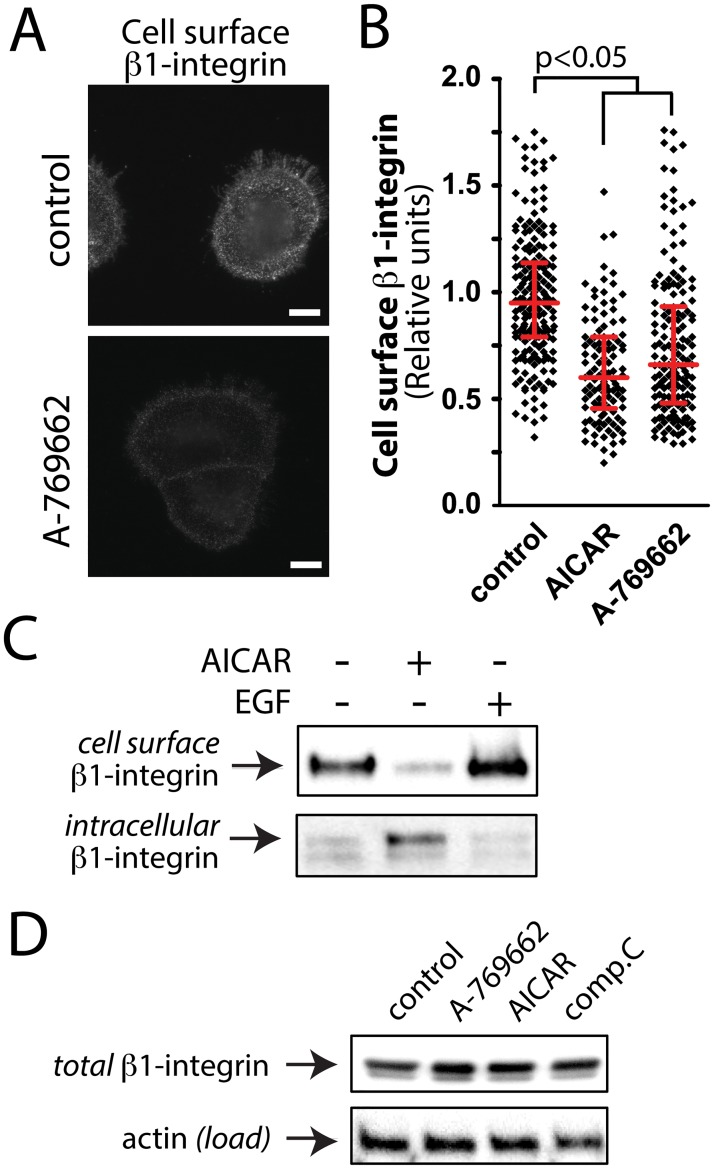
Treatment with A-769662 reduces cell surface β1-integrin levels. (***A***) RPE cells were stimulated with 100 μM A-769662 for 90 min or left unstimulated (basal). Intact cells were labeled with an antibody specific for an exofacial epitope on β1-integrin. Shown are representative fluorescence micrographs depicting cell surface β1-integrin fluorescence. Scale = 5 μm (***B***) Cell surface β1-integrin levels obtained by fluorescence microscopy were quantified as described in *Materials and Methods* and S5B Fig. Shown are the cell surface β1-integrin measurements in individual cells (diamonds) as well as the median ± interquartile range of these values in each treatment condition (n = 4 independent experiments). (***C***) RPE cells were stimulated with 2 mM AICAR for 90 min or left unstimulated (basal), followed by cell-surface biotinylation, purification of biotinylated proteins and immunoblotting of fractions with an antibody specific to β1-integrin. Shown is an immunoblot of cell surface β1-intergin (*top panel*, corresponding to the streptavidin pull-down), and of the corresponding intracellular β1-integrin (*bottom panel*, corresponding to the above supernatant), representative of 4 independent experiments. (***D***) Shown are representative immunoblots of whole-cell lysates prepared from cells stimulated with either 100 μM A-769662, 2 mM AICAR, 40 μM compound C (each for 90 min) or left unstimulated (control), probed with antibodies to detect total cellular β1-integrin or actin (load).

β1-integrin is the major binding partner for integrins-α4 and- α11, both of which were detected in the cell surface fraction of control but not A-769662 treated cells (Table 2). Thus, β1-integrin is a candidate target protein to be regulated by AMPK upon metabolic stress. To determine if AMPK activation indeed controls the cell surface abundance of β1-integrin, we measured cell surface β1-integrin levels in intact cells by immunofluorescence microscopy, using an antibody that detects an exofacial epitope on β1-integrin (Fig 4A), and activation of AMPK either by treatment with A-769662 or AICAR. We observed a decrease in cell surface β1-integrin fluorescence intensity in cells stimulated with 100 μM A-769662 compared to control cells. Quantification of the mean fluorescence intensity of cell surface β1-integrin fluorescence revealed a 25.1 ± 2.7% or a 36.3 ± 2.3% reduction in this parameter in cells treated with 100 μM A-769662 or 2 mM AICAR for 90 min to activate AMPK compared to control cells (n = 3, p < 0.05) **(** Fig 4B).

To complement the observations made by immunofluorescence microscopy, we examined cell surface β1-integrin levels using cell surface biotinylation and purification of biotinylated proteins, followed by western blotting with β1-integrin specific antibodies. Using this method, we observed that treatment with 2 mM AICAR (Fig 4C) or 100 μM A-769662 (S5D Fig) reduced β1-integrin cell surface levels compared to control, with a corresponding gain in intracellular β1-integrin (Fig 4C). In contrast to the effects observed with AMPK activation, treatment with 20 ng/mL epidermal growth factor (EGF) for 90 min had no effect on cell surface β1-integrin levels (Fig 4C).

Treatment with 100 μM A-769662, 2 mM AICAR, or 40 μM compound C (an AMPK inhibitor) for 90 min did not alter total cellular levels of β1-integrin (Fig 4D). This indicates that the decrease in cell surface β1-integrin upon treatment with 100 μM A-769662 occurred as a result in regulated changes in β1-integrin cellular localization and not as a result of changes in expression levels of this protein.

**Fig 5:**
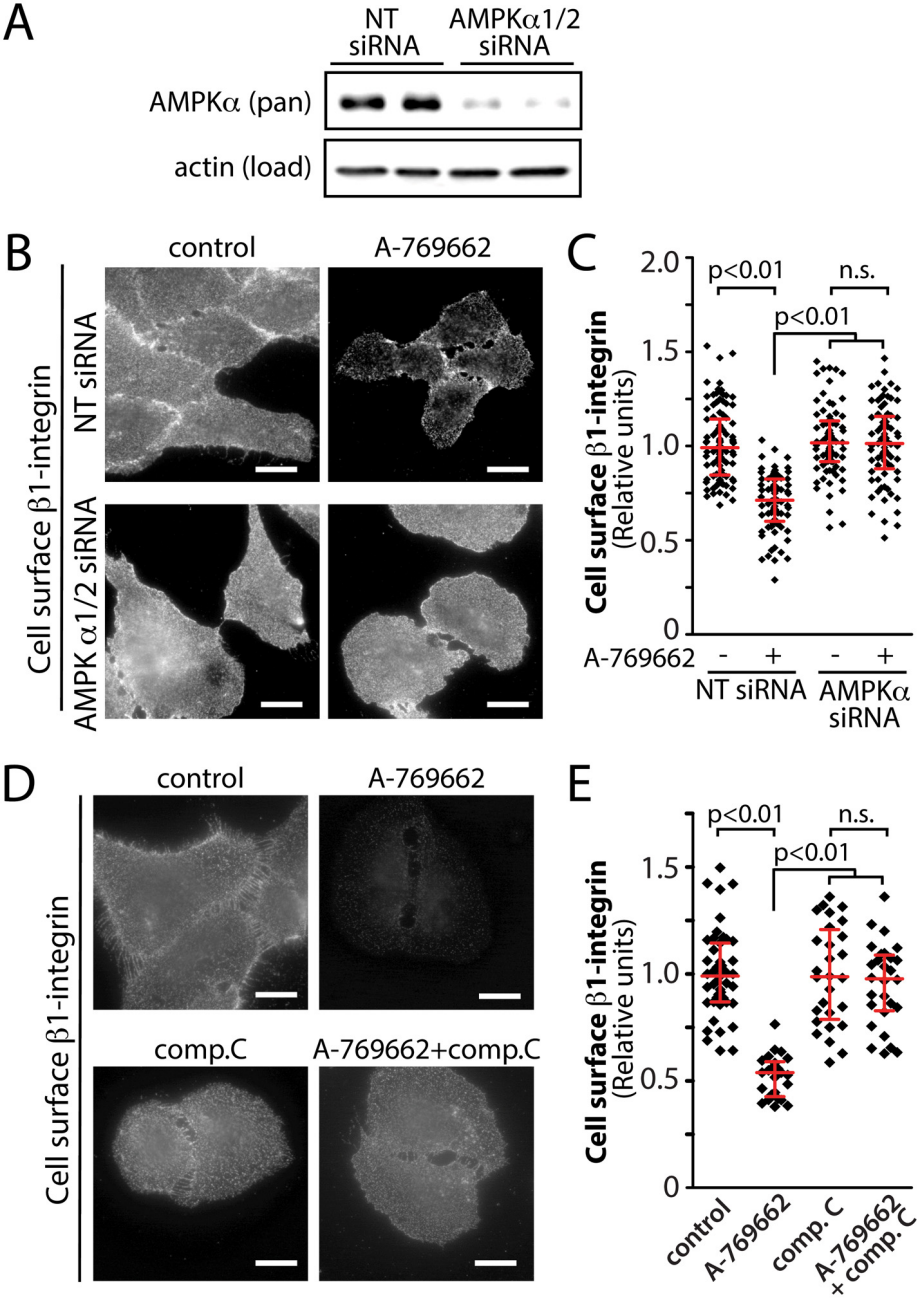
Inhibition of AMPK by siRNA gene silencing or by compound C prevents the reduction in cell surface β1-integrin elicited by A-769662 treatment. (***A-C)*** RPE cells were transfected with siRNA targeting AMPK α1/2 or non-targeting (NT, control) siRNA. (***A***) Whole cell lysates were prepared and resolved by immunoblotting and probed with anti-AMPK α1/2 or anti-actin antibodies. Shown are immunoblots representative of at least 3 independent experiments. (***B***) Following siRNA transfection, cells were treated with 100 μM A-769662 for 60 min as indicated. Intact cells were labeled with an antibody specific for an exofacial epitope on β1-integrin. Shown are representative fluorescence micrographs depicting cell surface β1-integrin fluorescence. Scale = 5 μm (***C)*** Cell surface β1-integrin levels obtained by fluorescence microscopy were quantified. Shown are the cell surface β1-integrin measurements in individual cells (diamonds) as well as the median ± interquartile range of these values in each treatment condition (n = 3 independent experiments). (***D***) RPE cells were treated with 100 μM A-769662 or 40 μM compound C, alone or in combination, for 60 min as indicated. Intact cells were labeled with an antibody specific for an exofacial epitope on β1-integrin. Shown are representative fluorescence micrographs depicting cell surface β1-integrin fluorescence. Scale = 5 μm (***E)*** Cell surface β1-integrin levels obtained by fluorescence microscopy as in (D) were quantified. Shown are the cell surface β1-integrin measurements in individual cells (diamonds) as well as the median ± interquartile range of these values in each treatment condition (n = 3 independent experiments).

To determine if treatment with A-769662 reduced cell surface β1-integrin as a result of activation of AMPK, we used two complementary strategies to perturb AMPK: siRNA gene silencing of AMPK and acute pharmacological inhibition with compound C. Treatment with siRNA targeting the α isoform of AMPK (both α1 and α2) resulted in a reduction of 69.6 ± 2.6% of AMPK α1/2 expression in RPE cells (n = 3) (Fig 5A). Consistent with results presented in Fig 4A, treatment of cells transfected with non-targeting (NT) siRNA with 100 μM A-769662 resulted in a 29.7 ± 1.7% reduction in cell surface β1-integrin levels (n = 3, p < 0.05) (Fig 5B and 5C). In contrast, cells transfected with AMPK α1/2 siRNA exhibited no change in cell surface β1-integrin upon similar treatment with A-769662 (Fig 5B and 5C). Consistent with these results, in the presence of compound C (an AMPK inhibitor), RPE cells exhibited no detectable change in cell surface β1-integrin upon treatment with 100 μM A-769662, while control cells (not treated with compound C) stimulated with A-769662 exhibited a 47.1 ± 2.9% reduction in cell surface β1-integrin levels (n = 3, p < 0.05) (Fig 5D and 5E). Thus, two independent methods of perturbation of AMPK (gene silencing and pharmacological inhibition) show that the reduction in cell surface β1-integrin by treatment with A-769662 requires active AMPK. Hence, AMPK activation controls cell surface β1-integrin abundance and membrane traffic.

**Fig 6:**
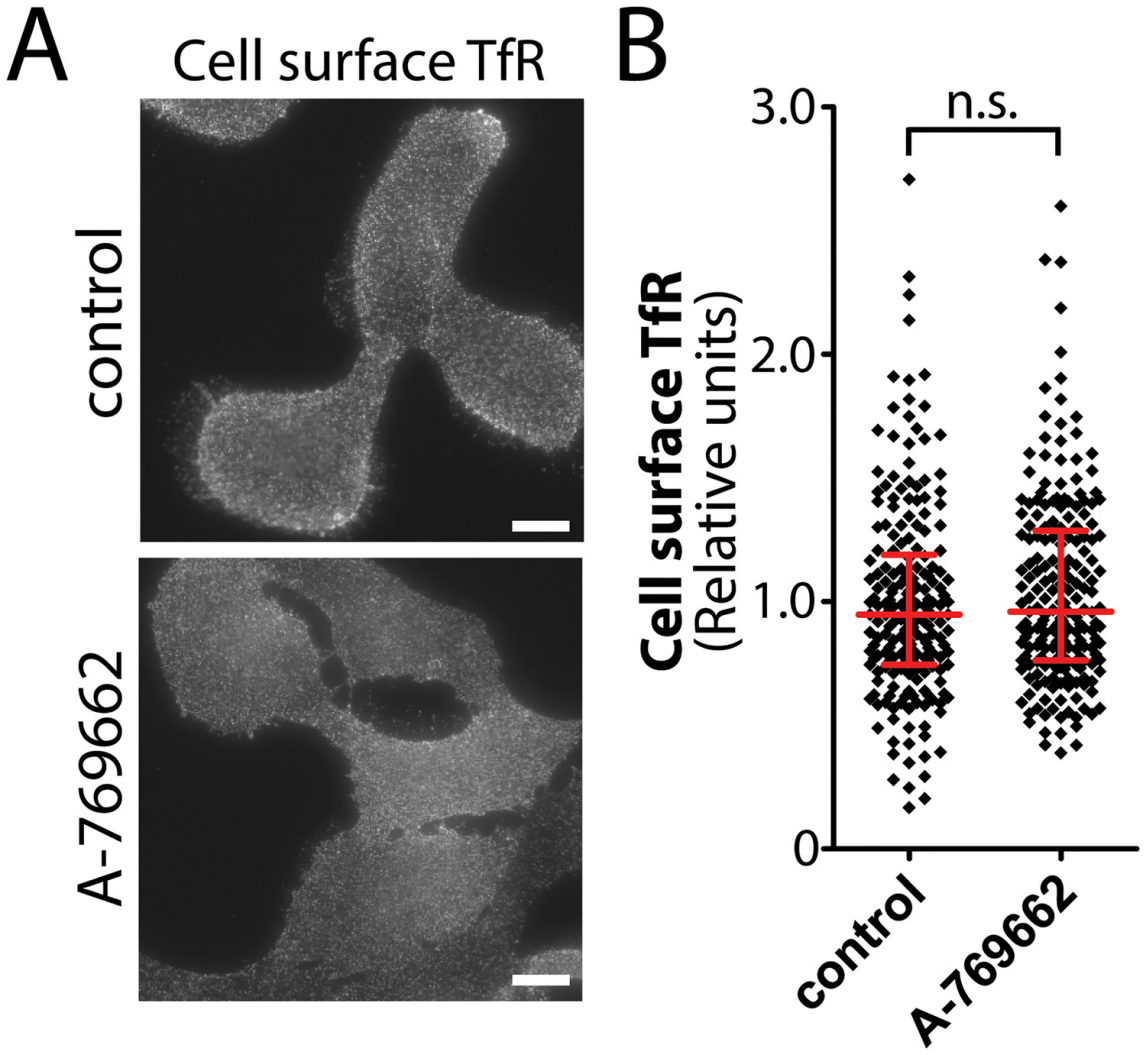
Treatment with A-769662 does not change cell surface TfR levels. (***A***) RPE cells were stimulated with 100 μM A-769662 for 90 min or left unstimulated (basal). Intact cells were labeled with an antibody specific for an exofacial epitope on Transferrin Receptor (TfR). Shown are representative fluorescence micrographs depicting cell surface TfR fluorescence. Scale = 5 μm (***B***) Cell surface TfR levels obtained by fluorescence microscopy were quantified. Shown are the cell surface TfR measurements in individual cells (diamonds) as well as the median ± interquartile range of these values in each treatment condition (n = 3 independent experiments).

Examination of the mass spectrometry data revealed that treatment with 100 μM A-769662 resulted in decreased cell surface detection of a large number of proteins, more than exhibited increased or unchanged detection upon this treatment (Fig 1B). This may reflect a robust and specific internalization of a large number of proteins under conditions of metabolic stress, or instead may reflect large-scale, rather non-specific membrane internalization triggered by A-769662 treatment. To distinguish between these possibilities, we measured the cell-surface levels of transferrin receptor (TfR), an abundant cell surface receptor that undergoes constitutive endocytosis and rapid recycling, using an antibody that detects an exofacial epitope of TfR. We observed no change in cell surface TfR fluorescence intensity in cells stimulated with 100 μM A-769662 compared to control cells (Fig 6A), a finding that was confirmed by quantification of cell surface TfR fluorescence intensity (n = 3) (Fig 6B). In addition, cell surface biotinylation coupled to western blotting revealed no change in TfR within the cell surface fraction upon A-769662 treatment (S5D Fig). Hence, treatment with 100 μM A-769662 does not cause non-specific bulk internalization of the plasma membrane, but instead may elicit specific control of cell surface membrane traffic of a large number of proteins.

Collectively, these results indicate that treatment with 100 μM A-769662 regulates membrane traffic for selective control of the cell surface levels of some proteins (e.g. reducing cell surface β1-integrin levels) but not others (e.g. TfR).

## Discussion

We performed a systematic analysis of the regulation of the cell surface proteome by AMP-activated protein kinase (AMPK) using the AMPK activator A-769662. We identified 838 proteins integral to or associated with the cell surface, of which 653 exhibit reduced detection within the cell surface fraction upon treatment with A-769662, and 93 which exhibit increased detection within the cell surface fraction upon treatment with A-769662 (Fig 1). Of the proteins that exhibit decreased detection at the cell surface upon A-769662 treatment, several GO functional terms are enriched: cell adhesion and migration, regulation of apoptosis, regulation of the actin cytoskeleton, and regulation of intercellular signaling (Table 1). We have validated the observations made by mass spectrometry with regards to the regulation of cell adhesion and migration by AMPK by showing that treatment with A-769662 indeed impaired cell migration in an epithelial wound-healing assay (Fig 3). Furthermore, we used immunofluorescence microscopy and western blotting, coupled with siRNA gene silencing and pharmacological inhibition of AMPK to validate the selective regulation of cell surface abundance of β1-integrin by AMPK (Figs 4–6).

### Coverage of the cell surface proteome

Our mass spectrometry identification of cell surface proteins likely underrepresents the complete cell surface proteome of RPE cells. This can be due to 1) the paucity of exposed lysine residues within either the exofacial or endofacial portion of some proteins, 2) the technical difficulties in achieving sufficient solubility of highly hydrophobic integral membrane proteins and 3) the stringency of our identification method, which we discuss below.

The sulfo-NHS-SS-biotin reagent is amine-reactive, and hence requires accessible extracellular amines for biotinylation of proteins. Furthermore, preparation of mass spectrometry samples involves tryptic digestion, which occurs on the carbonyl side of basic residues. Labeling of cell-surface proteins with the amine-reactive sulfo-NHS-SS-biotin effectively masks the positive charge on lysines, thus limiting tryptic cleavages sites of extracellular protein segments. For example, β1-integrin has a very large extracellular domain and a relatively small intracellular domain; as such, following cell surface biotinylation, β1-integrin is expected to have few available tryptic cleavage sites, which reduces the efficiency of identification of this protein.

Our protein identification regime was very stringent in order to eliminate false detection of background proteins. Firstly, we required a parent fragment intensity > 1,000 counts for each peptide. Further, we discounted protein identifications from the basal or A-769662 conditions (both treated with sulfo-NHS-SS-biotin) if any identification is also made from background samples (i.e. from cells not treated with sulfo-NHS-SS-biotin). As such, we discounted from our analysis proteins such as EGFR, β1-integrin, actin and proteins within the mitogen-activated protein kinase pathway (all observed by western blot to be present in the cell surface fraction, S1 Fig and Fig 4C) due to detection of corresponding peptides in the background sample (albeit a small number of such background peptides). Thus, while the remaining list of 838 cell surface proteins may underrepresent the cell surface proteome, it does represent high confidence identifications of specific cell surface proteins (peripheral or integral).

Some of the proteins that we have identified within the cell surface fractions had little available experimental information about their localization or function, but might be predicted to localize to membrane compartments other than the cell surface. For instance, ZNF142 harbors several C2 zinc finger domains that are typically found in DNA-binding transcription factors, yet was found at the cell surface in basal (74 peptides identified) but not A-769662-treated cells (0 peptides identified). Our examination of this protein in RPE cells using antibodies that can recognize the endogenous protein revealed localization mostly outside of the nucleus (S4A Fig). Importantly, ZNF142 exhibited peripheral, membrane-proximal localization in control cells, which was lost upon treatment with A-769662 (S4B Fig). Hence we were able to confirm using other methods that ZNF142 is associated with the cell surface fraction in control but not A-769662-treated cells. ZNF142 may indeed be a transcription factor that is retained in an inactive state in the cytoplasm or associated with the cell surface during some conditions. Alternatively ZNF142 may have functions exclusively outside of the nucleus. Consistent with the latter interpretation, some C2 zinc-finger domains can mediate protein-protein interactions instead of interactions with DNA [56].

### Regulation of the cell surface proteome by AMPK

Cells treated with A-769662 exhibited a broad reduction of detection of 653 proteins within the cell surface fraction. The decreased detection of proteins from the cell surface upon treatment with A-769662 may be in part due to regulation of the membrane traffic of intrinsic membrane proteins, along with their protein interaction networks. Consistent with this interpretation, of the 592 proteins depleted from the cell surface upon AMPK activation that had GO annotations, 79 were transmembrane proteins. AMPK is known to regulate the membrane traffic of some specific proteins, including that of the facilitative glucose transporters GLUT4 and GLUT1 and of the Na/K-ATPase [1].

GLUT1 internalization is enhanced by binding to the α-arrestin family protein, thioredoxin-interacting protein (TXNIP) [15]. Phosphorylation of TXNIP by AMPK results in its degradation, which in turn effects a reduced rate of GLUT1 endocytosis and enhanced rate of glucose uptake [15]. AMPK activation results in rapid internalization of the Na/K-ATPase, *via* the atypical Protein Kinase C-ζ -mediated phosphorylation of the pump [57], or the phosphorylation of the clathrin-mediated endocytosis adaptor protein AP-2 (μ2 subunit) [58]. The result of this regulation is increased energy intake (as a result of increased cell surface GLUT1) and decreased energy expenditure (as a result of decreased cell surface Na/K-ATPase), both of which contribute to cellular homeostasis under metabolic stress [1].

AMPK may broadly yet specifically regulate the cell surface proteome by control of α-arrestin family proteins [15] or by phosphorylation of AP-2 [58]. Indeed the amino acid metabolism sensor mTOR controls the plasma membrane content and endocytosis of several proteins *via* the kinase Npr1 and the arrestin-like protein Art1 in yeast [59]. As AMPK also controls the α-arrestin protein TXNIP, AMPK may broadly regulate the cell surface proteome by a mechanism analogous or similar to that of mTOR. In addition, AMPK activation regulates the Rab GTPases TBC1D1 and TBC1D4, which regulate the endomembrane traffic of GLUT4 [60]. As there are many members of the TBC1D family [61], broad regulation of the cell surface proteome by AMPK-dependent control of Rab GAPs and Rab-mediated intracellular membrane traffic is also an intriguing possibility. The control of endomembrane traffic by AMPK is broad yet at least partly selective, as cell surface levels of transferrin receptor (TfR) were not altered by A-769662 treatment (Fig 6).

Recent studies in *Saccharomyces cerevisiae* revealed that glucose starvation alters localization of clathrin adaptors to endosomes and to the trans-Golgi network (TGN), which was dependent on Snf1, the yeast homologue of the α-catalytic subunit of AMPK [62]. Glucose starvation reduces recycling to the plasma membrane, which causes a reduction in the cell surface abundance of proteins such as Mup1 [63]. Under glucose starvation conditions, decreased recycling leads to increased traffic of proteins to the vacuole, thus liberating amino acids to replenish cellular energy [63]. In RPE cells, activation of AMPK for 90 min did not alter cell surface TfR abundance, nor alter total cellular levels of β1-integrin (Fig 4D), suggesting mass routing of endomembranes to the lysosome did not occur during this time. The broad changes in the cell surface content of specific proteins observed in mammalian (RPE) cells upon AMPK activation may suggest some evolutionary conservation of energy stress signaling regulation of cell surface membrane traffic.

The change in detection of proteins within the cell surface fraction may also be due to alterations elicited by A-769662 treatment of the regulation of posttranslational modification of the cell surface proteome. As we are likely limited to detection of peptides corresponding to the intracellular segments of proteins (given that K-sites on ectodomains are inaccessible to trypsin cleavage once biotinylated), our identification of proteins may be particularly sensitive to reduced detection of cell surface proteins due to intracellular post-translational modifications. Indeed, our observation that the atypical cadherins FAT1 and FAT2 exhibit reduced abundance at the cell surface may reflect acute cleavage of the intracellular portions of these proteins to generate cytosolic fragments [64]. Such a regulated cleavage to remove the cytosolic portion of FAT would not impact cell-surface biotinylation but would limit the detection of FAT endodomain trypic fragments in the cell surface fraction. The cytosolic FAT fragment translocates to the mitochondria where it functions to promote oxidative production of ATP by direct binding of complex I [64]. As such, acute treatment with A-769662 to activate AMPK may be enhancing mitochondrial ATP production *via* cell-surface FAT-derived cleavage intermediates. Whether the altered detection of proteins in the cell surface fraction upon A-769662 treatment results from changes in membrane traffic or posttranslational modification, our results suggest a broad regulation of cell surface proteins as part of the AMPK-dependent cellular response to energy stress.

While treatment with A-769662 is one of the more selective methods to specifically activate AMPK [24,44,65], there have been several studies that have reported additional cellular effects of A-769662 treatment, including inhibition of the non-proteolytic components of the 26S proteasome [66], inhibition of the Na+/K+-ATPase [67], activation of Akt signaling [68], and alteration of the response to TRAIL ligand stimulation [69]. Despite these reports that some effects of A-769662 that are independent of AMPK, there are numerous studies that have indeed reported a wide range of cellular phenomena elicited by A-769662 that are dependent on AMPK, such as [24,44,70–72], including regulation of cell surface membrane traffic of GLUT1 [15]. A recent study revealed that while the AMPK activators AICAR, phenformin, metformin, 2-deoxyglucose and salicylate all exhibited numerous cellular effects that were insensitive to AMPK perturbation and thus likely represent off-target effects, A-769662 exhibited almost no such off-target effects [24]. Specifically, A-769662 treatment impaired mTOR signaling, proliferation (in media containing full serum), and oxygen consumption rate, all of which required AMPK [24]. Hence, A-769662 represents the best available tool for specific activation of AMPK and many of the cellular effects observed upon treatment with A-769662 are AMPK-dependent.

Consistent with this interpretation, using siRNA gene silencing and compound C to perturb AMPK, we find that the regulation of β1-integrin cell surface abundance by A-769662 treatment is indeed dependent on AMPK (Fig 5). Hence, our results indicate that the commonly used AMPK activator A-769662 elicits broad control of the cell surface proteome, and that at least some of this regulation is specifically due to AMPK activation by A-769662. It would nonetheless be advisable for any future studies examining the regulation of specific cell surface proteins by AMPK (e.g. as per S1 Table) to examine the mechanism by these proteins are impacted by A-769662 treatment.

Collectively, our mass spectrometry analysis together with additional methodologies allowed us to identify that the membrane traffic of β1-integrin is subject to control by AMPK.

### Regulation of cell migration and β1-integrin membrane traffic by AMPK

Epithelial cell migration is a critical cellular process involved in development, tissue homeostasis, and wound healing. Emerging evidence suggests that cellular energy stress may be a key regulator of cell adhesion and migration. Activation of AMPK by hypoxia reduces cell adhesion in endothelial progenitor cells [20]. Treatment of vascular smooth muscle cells with berberine (which activates AMPK) reduces cell migration in vascular smooth muscle cells [21], while with treatment with AICAR and phenformin [22] or metformin [23], all of which also activate AMPK, reduced cell migration in U937 monocytes and glioblastoma cells, respectively. As A-769662 is a more selective activator of AMPK than other agents [24], our observations that A-769662 treatment reduces cell migration provide important confirmation of a specific role for AMPK in the regulation of cell migration.

Furthermore, our mass spectrometry analysis and subsequent study of β1-integrin membrane traffic provides novel insight into the mechanism by which the regulation of cell migration by AMPK activation may occur. Specific β1-integrins heterodimers exhibit selectivity for binding to extracellular matrix (ECM) proteins, such as α1β1, α2β1 and α11β1 (to collagen), α5β1 and α4β1 (to fibronectin), and α3β1 and α6β1 (to laminin) [73]. Given that integrins α-11 and α-4 associate primarily with β1-integrin and both exhibited reduced detection in the cell surface fraction upon AMPK activation (Table 2), AMPK activation may elicit the internalization of several different integrin heterodimer types. Consistent with reduced cell surface β1-integrin and α-11 integrin, we observed reduced detection of 3 different types of human collagens (COL6, COL11 and COL14) within the cell surface fraction. As such, RPE cells may be undergoing β1-integrin-dependent phagocytic internalization of collagens (similar to that which occurs in fibroblasts [74]) upon AMPK activation.

The regulated membrane traffic of β1-integrin provides clues about the possible mechanism of regulation by AMPK. β1-integrin can undergo either clathrin-independent or dab2/ARH-dependent, clathrin-dependent [28] endocytosis. Upon internalization, β1-integrin localizes to specialized Rab21-positive early endosomes. β1-integrin is retained in Rab21-endosomes by interaction with Rab21 [75]. P120RasGAP displaces the Rab21/integrin interaction and is required for β1-integrin exit from Rab21-endosomes [76]. β1-integrin recycling and membrane traffic also requires GTPase-active Arf6 and Rab11 [30,31,77]. Thus, AMPK may be regulating one or several of these membrane traffic stages of β1-integrin.

In addition, there is well-described and close interplay between integrins and the actin cytoskeleton [78]. Given the reduced detection of cytoskeletal proteins in the cell surface fraction upon the internalization of integrins observed upon A-769662 treatment (Table 1), the reduced cell surface β1-integrin levels may also reflect control of the cortical or lamellipodial actin network by AMPK. Indeed, perturbation of AMPK through siRNA gene silencing or inhibition by compound C resulted in inhibition of cell migration, and robust changes in microtubule dynamics and actin polymerization, an effect that was entirely ascribed to the phosphorylation of the microtubule capping protein CLIP-170 by AMPK [11]. However, in this previous study [11], while AMPK inhibition or silencing reduced the phosphorylation of CLIP-170, activation of AMPK by AICAR had no effect on CLIP-170 phosphorylation [11]. Hence, it is unlikely that the changes in cell surface abundance of adhesion and migration proteins that we observe here upon AMPK activation are due solely to changes in CLIP-170 phosphorylation. Interestingly, both treatment with the AMPK activator A-769662 (this study, Fig 3) and silencing or inhibition of AMPK [11] each impair cell migration, suggesting that AMPK exerts tight control of cell migration by multiple mechanisms.

### Other proteins and processes regulated by AMPK

That the majority of cell surface proteins that we have identified (in all conditions) exhibit reduced detection within the cell surface fraction upon A-769662 treatment may reflect reduced energy expenditure by selective down-regulation of energy-consuming processes or adaptive responses to increase energy intake. While we have here focused on further characterizing the regulation of cell surface proteins involved in control of cell migration and adhesion, there are many proteins that exhibit previously unappreciated regulation of cell surface abundance upon treatment with A-769662. While beyond the scope of this study, future work examining in detail the molecular mechanisms and physiological outcomes of the control of the endomembrane traffic and/or association with the cell surface of many of these proteins by AMPK will provide valuable information about the control of cell physiology by metabolic signals. Importantly, drugs such as metformin (an AMPK activator) have long been used to treat type II diabetes and targeting of AMPK is emerging as a promising anti-cancer therapeutic strategy. Hence, a better understanding how AMPK controls cell physiology, in particular relating to the control of cell surface proteins and functions such as cell adhesion and migration such as revealed here, will enhance the effectiveness of the use of these therapies.

In conclusion, we have identified that treatment with the AMPK activator A-769662 exhibits broad yet specific control of the cell surface proteome. We have also found that treatment with A-769662 activation impairs cell migration, and that A-769662 reduces cell surface abundance of specific integrins in a manner that requires AMPK. This indicates that AMPK activation during conditions of energetic stress elicits an adaptive response to reduce energy consumption by halting the energy-intensive process of cell migration.

## Materials and Methods

### Materials

A-769662 was obtained from Abcam (Cambridge, MA), AICAR was obtained from Cell Signaling Technology (Danvers, MA). Sulfo-NHS-SS-biotin was obtained from Pierce (Thermo Fisher Scientific, Rockford, IL). Antibodies used for immunoblotting were as follows: anti-EGFR from Genetex (Irvine, CA), anti-CHC from Santa Cruz Biotechnology (Santa Cruz, CA), anti-pACC, anti-AMPK (α1/2), anti- actin, and anti-Erk from Cell Signaling Technology (Danvers, MA), and anti-ZNF142 antibodies from Aviva Systems Biology (San Diego, CA). Antibodies used for immunofluorescence microscopy were as follows: anti-β1-integrin from EMD Millipore, Darmstadt, Germany), and anti-TfR from Santa Cruz Biotechnology (Santa Cruz, CA).

### RPE Cell Culture and pharmacological AMPK activation or inhibition

Human non-immortalized Retinal Pigment Epithelial (ARPE-19) cells were obtained from ATCC (henceforth RPE cells). All RPE cells were maintained in in DMEM F12 supplemented with 10% fetal bovine serum (FBS) and 5% streptomycin/penicillin in a humidified incubator at 37°C and 5% CO_2_. For experiments requiring AMPK activation, RPE cells were incubated in low serum media (0.1% FBS in DMEM F12) for one hour, after which they were treated with 100 μM A-769662 or 2 mM AICAR as indicated (while remaining in the 0.1% FBS DMEM F12 media) to activate AMPK. In some experiments (Figs 4D and 5), cells were also treated with 40 μM compound C during treatment with AMPK activators.

### Cell Surface Protein Labelling and Purification

In order to selectively and covalently modify cell surface proteins with a biotin moiety, cells were ectopically labelled with 0.5 mg/mL Sulfo-NHS-SS-biotin in PBS for 30 min at 16°C and then quenched with 50 mM Tris-HCl for 10 min at 16°C. Cells were then homogenized in modified RIPA buffer (50 mM Tris-Cl pH 7.4; 0.25% Na deoxycholate; 150 mM NaCl, 1 mM EDTA; 50 mM n-octylglucoside) supplemented with protease inhibitors. Homogenized cells were incubated under constant rotation at 4°C for 2 h and then centrifuged at 13000 rpm for 15 minutes. After centrifugation, the pellet was discarded and the protein concentration was measured. Equal amounts of protein were incubated under constant rotation with streptavidin-conjugated sepharose beads overnight at 4°C. Intracellular proteins were collected in the supernatant and after rigorous RIPA washes, cell-surface proteins were eluted with 100 mM dithiothreitol (DTT).

### Mass spectrometry and GO Analysis

Cell-surface proteins were incubated with digestion buffer (1 M Tris; 2 M Urea; 50% Acetonitrile; pH 8.8) and 1 μL trypsin (Roche, Indianapolis, IN) at 37°C overnight. Samples were incubated in 2 mM DTT for 1h at 50°C. After being brought to room temperature, samples were incubated with 1 μL of trypsin (Roche, Indianapolis, IN) for 1h at 37°C followed by 5% formic acid solution. LC-ESI-MS/MS analysis was performed after solid-phase extraction of peptides using Zip-Tip C18 (EMD Millipore, Billerica, MA) stationary phase for small-scale sample cleanup, as previously described [79].

LC-ESI-MS/MS was performed using a reversed phase column (15 cm, 300 μm ID) packed with C18 beads of 5 μm diameter and 300-Å pore size equipped with an Agilent 1100 HPLC pump, followed by analysis using an LTQ XL ion trap (Thermo Electron Corporation, Waltham, MA.). Parent peptide fragments required a minimum of 1000 counts to be analyzed further. Data for charge states 2+, 3+, with a minimum peptide length of 5 were run against federated library of all human proteins using the search algorithms SEQUEST, TANDEM, MASCOT, and OMSSA set for fully tryptic peptides with a maximum 2 missed cleavages to identify proteins, as previously described [51–53]. All proteins that had at least 1 peptide identification in background samples were subtracted from the total list of results. The total 838 cell surface proteins were classified into three categories: (i) “depleted from the cell surface in A-769662 cells” if they were detected by a minimum of 4 peptides in the basal condition and no (0) peptides in the A-769662 condition, (ii) “enriched at the cell surface in A-769662 cells” if they were detected by a minimum of 4 peptides in the A-769662 condition and no (0) peptides in the basal condition, and (iii) “unchanged in cell surface abundance in A-769662 cells”, if peptides corresponding to the protein were detected in both basal and A-769662 conditions (see S1 Table). GO annotation terms enriched in each of the 3 categories was determined using DAVID bioinformatics resources (version 6.7, see http://david.abcc.ncifcrf.gov/summary.jsp) [50]. The parent and fragment ion intensity values were analyzed using goodness of fit and ANOVA using SQL and R after the method of Florentinus et al. [49].

The mass spectrometry data is compliant with the Minimum Information About a Proteomics Experiment (MIAPE) standards [80,81]; this data is stored in an SQL database that includes for parent ions: m/z, peptide charge, number of missed cleavages; and for MS/MS fragments: m/z of parent ion, m/z of each fragment and fragment intensity. A sample of this data is provided in S4 Table and access to the complete SQL database is readily provided upon request.

### Immunoblotting

Whole-cell lysates were prepared in Laemmli Sample Buffer (LSB, 0.5M Tris pH 6.8, Glycerol, 10% SDS, 10% β-mercaptoethanol, and 5% bromophenol blue, all from BioShop, Burlington, ON) supplemented with a protease and phosphatase cocktail (1 mM sodium orthovanadate, 10 nM okadaic acid, and 20 nM Protease inhibitor cocktail (BioShop, Burlington, ON). Lysates were then heated at 65C for 15 min and passed through a 27.5 gage syringe.

Lysates from whole-cell extracts, cell-surface fraction, or intracellular fraction were probed by Western blotting as previously described [82]. Briefly, samples were subjected to gel SDS-PAGE, transferred to PVDF membranes (Bio-Rad, Burlington, ON). The membranes were blocked in a solution of TBS-T containing 4% BSA or 3% milk for 30 minutes at room temperature prior to incubation with appropriate antibodies and ECL detection.

### Cell Migration Assay

To initiate the cell migration experiment, RPE cells grown to confluence in a 35 mm dish with a grid (Sarstedt Canada, Montreal, QC) were wounded by a single passage of a P10 micropipette tip. Cells were then immediately washed and placed in media containing 0.1% FBS (to minimize cell proliferation), either also supplemented with 100 μM A-769662 or not (control). Images were acquired at 0 and 24 hours following wounding. Images of cells were acquired using a Leica DM IL microscope equipped with a Skylight camera phone microscope mount, using a Samsung Galaxy S4 smartphone digital camera. Images were manually aligned using dish grid. Cell migration was quantified by measuring the area of the wound covered by cells after 24 h (using manual delineation of cell coverage area using Image J), and expressed as a percent of the initial wound area. At least 20 individual wounded areas were examined for each condition in each experiment.

### siRNA transfection

RPE cells were transfected with siRNA using custom-synthesized siRNAs using RNAiMAX transfection reagent (Life Technologies, Carlsbad, CA), as per manufacturer’s instructions. Briefly, each siRNA was transfected at a concentration of 220 pmol/L with the transfection reagent in Opti-MEM media (Life Technologies, Carlsbad, CA) for 4 hours, followed by washing and replacement with regular growth media. siRNA transfections were performed twice (72 h and 48 h) prior to each experiment. Sequences used were as follows: non-targeting (NT) control: (sense) CGUACUGCUUCGGAUACGGUU, (antisense) CCGUCUCGCAAGCAGUACGUU and AMPK α1/2: (sense) GCACCUUCGGCAAAGUGAAUU, (antisense) UUCACUUUGCCGAAGGUGCUU (Dharmacon, GE Healthcare, Lafayette, CO).

### Fluorescence microscopy and cell surface protein abundance quantification

Immunofluorescence detection of cell-surface β1-integrin and transferrin receptor levels. Cell surface proteins were detected as previously described [13]. Briefly, intact cells grown on coverslips were blocked for 15 minutes on ice (to arrest membrane traffic) in a solution of PBS+ containing 3% BSA, followed by labeling with a solution containing an antibody to detect an exofacial epitope (of TfR or β1-integrin) for 1h at 4°C. Cells were then washed extensively, fixed in a solution of 4% PFA, followed by quenching of the fixative in a 100 mM glycine solution, and detection of surface-bound primary antibodies with the appropriate secondary antibodies. After extensive washing, coverslips were mounted in Dako fluorescent mounting media (Dako, Carpinteria, CA).

Immunofluorescence detection of total cellular ZNF142. Intact RPE cells stably expressing clathrin light chain fused to GFP (GFP-clathrin, [43]) and grown on coverslips were treated as indicated and then fixed in a solution of 4% PFA. Subsequently, cells were quenched in a solution of 100 mM glycine, following by blocking in a solution of containing 3% BSA. Then, cells were labelled sequentially with anti-ZNF142 primary antibodies and appropriate secondary antibodies. After extensive washing, coverslips were mounted in Dako fluorescent mounting media (Dako, Carpinteria, CA).

Cell viability determination by propidium iodide. Following cellular treatment as indicated, intact RPE cells were incubated with 500 nM propidium iodide for 15 min at 4C, followed by washing, fixation and mounting in Dako fluorescent mounting media as described above. Parallel samples (positive control for propidium iodide staining) were first subjected to fixation (4% PFA for 30 min) and permeabilization (0.1% TX-100 for 20 min) prior to labeling with 500 nM propidium iodide.

Immunofluorescence microscopy was performed using a 63x (NA 1.49) oil objective on a Leica DM5000 B epifluorescence microscope using a DFC350FX camera (Leica Microsystems, Wetzlar, Germany). Images were acquired using Adobe Photoshop (San Jose, CA) and all exposure times and image scaling were equal within an experiment. Surface β1-integrin or TfR in each cell was quantified using ImageJ software (National Institutes of Health, Bethesda, MD) [83], as previously described [13]. Delineation of cellular contour was readily visible in each cell; regions of interest corresponding to cellular contour (whole cell) were manually delineated in Image J, and the mean pixel intensity within each cell was determined (S5B Fig, *red traces*). Background fluorescence values were determined by subtraction of the mean pixel intensity of a region of interest on the coverslip outside of the cell area (S5B Fig, *yellow traces*). Similar measurements were performed on cells subjected to a similar immunofluorescence method but without incubation with specific primary antibodies. Cell surface secondary antibody labeling in cells not incubated with primary antibody was ~10% of that of control cells incubated with either anti- β1-integrin or-TfR antibodies; the mean no-primary fluorescence intensity value were subtracted from all conditions in each experiment. Treatment with A-769662 did not change cell area (S5C Fig), indicating that the changes in cell surface β1-integrin measured by quantification of mean fluorescence intensity was indeed caused by changes in the abundance of β1-integrin at the cell surface by this treatment.

Images were 16-bit and typical intensity ranges were between 10–30000 units ensuring pixel intensity saturation did not occur. Measurements of β1-integrin cell surface abundance under each set of conditions were subject to one-way analysis of variance (ANOVA) with Newman-Keuls post-test, with p < 0.05 as a threshold for significant difference among conditions. Measurements of TfR cell surface abundance under two conditions (Control, A-769662) were subject to a Student’s t-test, with a p < 0.05 as a threshold for significant difference among conditions.

## Supporting Information

S1 Table Shown is the complete list of proteins identified as (i) depleted from the cell surface upon A-769662 treatment, (ii) enriched at the cell surface by A-769662 treatment and (iii) largely unchanged at the cell surface by A-769662. (shown in attached. xls document)
(XLSX)

S2 Table Shown are the mean log intensity values of peptide fragments detected from a subset of proteins (FAT1, FAT2, COL6A3, COL14A1, ITGA11, ITGA4, ICAM5) identified in the cell surface fraction in the basal condition but not in A-769662-treated or background cell surface fractions. Also shown are mean log intensity values for peptide fragments from proteins detected only in the cell surface fraction of A-769662-treated cells but not control or background cell surface fractions (MYH13, PDZD2), proteins detected in the cell surface fraction of both control and A-769662-treated cells but not background samples (DNAH5, VAC14) and proteins detected in all three fraction (MCAF1, TLN1). n.d. = not detected.
(PDF)

S3 Table Shown are the total mean intensities of parent ions and MS/MS fragments for the subset of proteins listed in S2 Fig. The total parent ion intensities and the MS/MS fragment ion intensities were very similar between control and A-769662 treated cells, showing that the differences in detection of specific proteins between conditions was unlikely to be due to sampling error.
(PDF)

S4 Table Shown are sample mass spectrometry measurements for a subset of peptides corresponding to integrin α-11. Shown are the following for parent ions (first tab): *m/z*, peptide charge, number of missed cleavages; and for MS/MS fragments (second tab): *m/z* of parent ion, *m/z* of each fragment and fragment intensity (shown in attached. xls document).
(XLSX)

S1 Fig Cell surface biotinylation allows selective purification of integral and membrane-associated cell surface proteins. RPE cells were subjected to surface biotinylated by treatment with sulfo-NHS-SS-biotin or left untreated (background), following by purification of cell surface proteins by streptavidin bead pull-down. (***A***) Shown is a representative silver-stained gel of cell surface fractions from (i) non-biotinylated (Background) or (ii and iii) sulfo-NHS-SS-biotin-treated cells. (***B***) Shown are representative immunoblots using anti-EGFR, anti-clathrin-heavy chain (CHC), anti-Erk or anti-actin specific antibodies following streptavidin pull-down using a range of cell lysate concentrations from each condition as input. The pull-down corresponds to the cell surface fractions and the s.n. (supernatant) corresponds to the non-biotinylated intracellular fractions.
(EPS)

S2 Fig Peptide spectra obtained by mass spectrometry. Shown are sample MS/MS spectra for various peptides identified in this study. The spectra indicate that many MS/MS fragments fit the peptide model attributed to the parent ion within 0.5 Da that were above the background noise.
(EPS)

S3 Fig ZNF142 is largely localized outside of the nucleus and detection at the cell periphery is regulated by AMPK activation. RPE cells stably expressing clathrin light chain fused to GFP (GFP-clathrin) were stimulated with 100 μM A-769662 for 90 min or left unstimulated (basal). Following fixation and permeabilization, cells were labeled with an antibody specific for ZNF142. (***A***) Shown are representative fluorescence micrographs depicting total cellular ZNF142, GFP-clathrin fluorescence or DAPI, as indicated. Scale = 20 μm. (***B***) Shown are magnifications of the boxed area within merged images shown in (A). Arrows indicate cell periphery, which exhibits visible enrichment of ZNF142 in basal (unstimulated) cells, but not in cells stimulated with A-769662.
(EPS)

S4 Fig Parent ion and MS/MS fragment intensities exhibited a mostly log-normal distribution. (***A***) Shown is a graph of the distribution of parent ion log intensities detected for the subset of proteins listed in S2 Table (black open circles). (***B***) Shown is a graph of the distribution of MS/MS fragment intensities for integrin α-11 (black open circles). For both (A) and (B), also shown in red is the ideal log-normal distribution.
(EPS)

S5 Fig Treatment with AMPK activators does not alter cell viability yet regulates cell surface β1-integrin levels. (***A***) RPE cells were treated using identical conditions as used for the cell migration assay (Fig 3, 0.1% FBS in DMEM with 100 μM A-769662). Following this treatment, intact cells were subjected to staining using propidium iodide (PI); cells prepared in parallel that were first fixed and permeabilized prior to treatment with PI provide an effective positive control for PI staining to detect permeabilized (non-viable) cells. Scale = 5 μm. (B) Shown are representative micrographs similar to those shown in Figs 4 and 5 of β1-integrin cell surface labeling by immunofluorescence. Shown overlaid on the right panel are regions of interest delineating the cell periphery (1–2) as well as background regions of the coverslip (3), used for measurement of cell surface β1-integrin levels as described in the Materials and Methods. (***C***) Using the method described in (B), cell area in control and A-769662-treated cells was determined. Shown are the values of cell area (in pixels, all images acquired in the same manner) obtained for individual cells (diamonds), as well as the median ± interquartile range of these values in each treatment condition. (***D***) RPE cells were stimulated with 2 mM AICAR or 100 μM A-769662 for 90 min or left unstimulated (basal), followed by cell-surface biotinylation, purification of biotinylated proteins and immunoblotting of cell surface fractions. Shown is an immunoblot of cell surface β1-intergin or cell surface transferrin receptor (TfR).
(EPS)
