# Supplementary material for: AMP-Activated Protein Kinase Regulates the Cell Surface Proteome and Integrin Membrane Traffic
Source: PLoS One. 2015 May 26;10(5):e0128013. doi: 10.1371/journal.pone.0128013 (PMC4444004; doi:10.1371/journal.pone.0128013)
Supplement: S2 Table — Also shown are mean log intensity values for peptide fragments from proteins detected only in the cell surface fraction of A-769662-treated cells but not control or background cell surface fractions (MYH13, PDZD2), proteins detected in the cell surface fraction of both control and A-769662-treated cells but not background samples (DNAH5, VAC14) and proteins detected in all three fraction (MCAF1, TLN1). n.d. = not detected. (PDF) [file pone.0128013.s002.pdf]

SUPPLEMENTAL TABLE 2

Ross et al. 2015

Gene sym.	Protein description	Mean Fragment intensity value		
		Basal	*A-769662 treated*	*Background*
FAT1	FAT tumor suppressor 1	0.728 ± 0.024	n.d.	n.d.
FAT2	FAT tumor suppressor 2	0.795 ± 0.035	n.d.	n.d.
COL6A3	collagen, type VI, alpha 3	0.855 ± 0.009	n.d.	n.d.
COL14A1	collagen, type XIV, alpha 1	0.856 ± 0.028	n.d.	n.d.
ITGA11	integrin, alpha 11	0.869 ± 0.013	n.d.	n.d.
ITGA4	integrin, alpha 4	0.938 ± 0.041	n.d.	n.d.
ICAM5	intercellular adhesion molecule 5	0.860 ± 0.011	n.d.	n.d.
MYH13	Myosin heavy chain 13	n.d.	0.772 ± 0.022	n.d.
PDZD2	PDZ domain containing 2	n.d.	0.731 ± 0.037	n.d.
DNAH5	Dynein heavy chain 5	0.547 ± 0.030	0.710 ± 0.037	n.d.
VAC14	ArPIKfyve	1.295 ± 0.029	1.047 ± 0.017	n.d.
TLN1	Talin 1	0.694 ± 0.013	1.235 ± 0.048	0.887 ± 0.012
MCAF1	Act. Trans. Fact. 7 Interact. Protein	0.661 ± 0.005	0.647 ± 0.006	0.642 ± 0.004
