# Supplementary material for: AMP-Activated Protein Kinase Regulates the Cell Surface Proteome and Integrin Membrane Traffic
Source: PLoS One. 2015 May 26;10(5):e0128013. doi: 10.1371/journal.pone.0128013 (PMC4444004; doi:10.1371/journal.pone.0128013)
Supplement: S3 Table — The total parent ion intensities and the MS/MS fragment ion intensities were very similar between control and A-769662 treated cells, showing that the differences in detection of specific proteins between conditions was unlikely to be due to sampling error. (PDF) [file pone.0128013.s003.pdf]

SUPPLEMENTAL TABLE 3

Ross et al. 2015

Treatment condition	Mean log intensity
Parent ions	
Basal	3.728 ± 0.029
A-769662 treated	3.800 ± 0.037
Background	3.938 ± 0.026
MS/MS fragments	
Basal	0.712 ± 0.004
A-769662 treated	0.721 ± 0.005
Background	0.673 ± 0.004
